# Oncologist responses to advanced cancer patients’ lived illness experiences and effects: an applied conversation analysis study

**DOI:** 10.1186/s12904-022-00917-4

**Published:** 2022-03-17

**Authors:** Jacqueline van Meurs, Wyke Stommel, Carlo Leget, Joep van de Geer, Evelien Kuip, Kris Vissers, Yvonne Engels, Anne Wichmann

**Affiliations:** 1 Department of Spiritual and Pastoral Care & Department of Anesthesiology, Pain and Palliative Medicine, Radboud University Medical Centre, P.O. Box 9101 (714), Nijmegen, 6500 HB Netherlands; 2 Centre for Language Studies, Radboud University, Nijmegen, the Netherlands; 3 Department of Care and Welfare, University of Humanistic Studies, Utrecht, The Netherlands; 4 Academic Hospice Demeter, Bilthoven & Agora, De Bilt, the Netherlands; 5 Department of Medical Oncology & Department of Anesthesiology, Pain and Palliative Medicine, Radboud University Medical Centre, Nijmegen, the Netherlands; 6 Department of Anesthesiology, Pain and Palliative Medicine, Radboud University Medical Centre, Nijmegen, the Netherlands

**Keywords:** Advanced cancer, Palliative care, Communication, Lived illness experience, Oncologists, Outpatients

## Abstract

**Background:**

An advanced cancer patient’s life is often disturbed by fear of cancer recurrence, cancer progress, approaching suffering, and fear of dying. Consequently, the role of the medical oncologist is not only to provide best quality anti-cancer treatment, but also to address the impact of disease and treatment on a patient’s life, the lived illness experience. We aimed to gain insights into whether and how medical oncologists working at an outpatient clinic identify and explore lived illness experiences raised by patients with advanced cancer, and how this influences patients’ responses.

**Methods:**

Conversation Analysis was applied to analyse 16 verbatim transcribed audio-recorded consultations.

**Results:**

We identified 37 fragments in which patients expressed a lived experience from 11 of the 16 consultations. We found differing responses from different oncologists. Patients continued talking about their lived experiences if the listener produced a continuer such as humming or tried to capture the experience in their own words. In contrast, a response with optimistic talking or the presentation of medical evidence prevented patients from further unfolding the experience. In consultations in which the lived illness experience was most extensively unfolded, medical oncologists and patients could constantly see each other’s facial expressions.

**Conclusions:**

When a patient with advanced cancer spontaneously introduces a lived illness experience, it helps to identify and explore it when the medical oncologist produces a continuer or tries to capture this experience in their own words.

Our findings can be implemented in training sessions, followed by frequent reinforcement in daily care.

**Supplementary Information:**

The online version contains supplementary material available at 10.1186/s12904-022-00917-4.

## Background

In recent decades, life expectancy of patients with advanced cancer has increased significantly as a result of innovations in diagnostics and treatments. However, their life is often disturbed by fear of cancer recurrence, cancer progress, approaching suffering, and fear of dying [1, 2].

Consequently, the role of the medical oncologist is not only to provide best quality anti-cancer treatment, but also to address the impact of the disease and its treatment on a patient’s life, the lived illness experience [3–5]. Considering the uniqueness of patients’ illness experiences, medical oncologists should, as stated in the WHO definition of palliative care, not only pay attention to the somatic, but also to the social, psychological and spiritual aspects of being ill [6, 7]. Knowing what matters most to the patient is a prerequisite for successful personalized care [5, 8, 9], and patients and their proxies confronted with a life-threatening disease highly value attention given by healthcare providers’ [10, 11].

For several reasons, lived illness experiences are underexplored. Patients with advanced cancer, even in the last months of life, often find it difficult to acknowledge that they will not be cured, which hampers open communication [12]. Moreover, not all patients are capable of explicitly articulating what is on their mind [10]. Often, attention for their experiences and concerns is asked implicitly, and recognizing these signals can be challenging [13, 14]. Even though clinicians receive communication training during their education and specialisation, training in signalling and exploring lived illness experiences is scarce [3, 15]. Moreover, clinicians consider responding to patients’ lived illness experiences, if signalled, time-consuming [16], even though it has shown that it doesn’t increase consultation length [17].

A recent review covering deficiencies in the current state of communication between medical oncologists and patients living with serious illness concluded that it is one of the most important ways that clinicians influence patient-centred care [18]. A JAMA consensus conference paper highlighted the need for research to improve quality of communication between health care professionals and patients living with serious illness [19]. However, there is a lack of insights in what happens in usual medical oncologists – patient conversations concerning lived illness experiences. Therefore, we explored whether and how medical oncologists, during their daily consultations, identify and explore lived illness experiences raised by their patients with advanced cancer.

## Methods

### Participants and procedure

This observational study took place at a university medical centre in the Netherlands. Between January and March 2019, outpatient consultations with medical oncologists and patients with advanced cancer with the outcome of a scan communicated were observed and audio-recorded by JvM, who previously worked with participating oncologists as a spiritual caregiver. In addition, field notes were made on non-verbal communication, including drawings depicting positions of physician, patient, relative and computer screen.

The oncologists were informed about the research project orally and by email. Patients with advanced cancer were approached by a nurse. They received information about the study, the voluntary nature of participation, and were able to ask questions. Participants gave their written informed consent. An exclusion criterion was a patient aged under 18. Data collection was stopped when central trends were identified and confirmed in further analysis.

### Design

We chose to analyse the data according to ‘applied conversation analysis’, an applied form of the classical method of conversation analysis (CA). CA explores the organization of naturally occurring talk, like medical consultation, and focusses on what the interlocutors are doing when talking, for example activities like turn-taking or self-repair of what has been said [20, 21]. Within CA, the researcher does not intervene in the interactions and is only present for the technical execution of the recording [21]. In classical CA, the analysis of data is done through “unmotivated viewing”, or “by approaching the data with nothing special in mind” [22]. Applied CA however, provides room for a more practical approach towards a direct question or problem [23]. The applied approach was used as this study aimed to gain insights into whether and how the medical oncologists, during their daily consultations, identify and explore lived illness experiences raised by their patients.

### Data analysis

Characteristics of patients and oncologists (supplemental material) were collated using descriptive statistics. The COREQ checklist for observational research guided the reporting.

#### Building collection

Audio-recordings of the consultations were listened to by three researchers (ABW, JvM, YE) several times, transcribed verbatim, and the transcriptions were read and reread. Two researchers (JvM,& ABW) independently searched the transcripts for fragments in which the patient raised a lived illness experience, such as being happy or worried (*‘I am very happy because I was worrying a bit….’)*, or by talking about fears (‘it is tense though’) or hope (*‘I hope I can still make it’*). A collection of these fragments was critically read by and discussed with a team of experts consisting of a medical oncologist, a patient diagnosed with incurable cancer, a general practitioner, and a spiritual caregiver [24]. On agreement, stretches of this collection were re-transcribed according to Jeffersonian transcription conventions (Table 1) to capture details such as laughter, overlapping talk, length of silences, inhalations and exhalations, sound stretches, faster and slower speech etc. [24].

**Table 1:** Jeffersonian transcription conventions

(.)	Short, untimed pause
(1.4)	Timed pause
hh	Exhalation
.hh	Inhalation
(word)	Unclear hearing
((comment))	Transcriber’s comment
w[ord	Overlapping onset
wor]d	Cut-off word
wor-	Faster speech rate
>word<	Slower speech rate
↓word	Markedly lower pitch
↑word	Markedly higher pitch
word=	Latching, rush into next turn or segment
word	Prominent stress
WORd	Higher volume than surrounding talk
wo:rd	Lenghtening of segment
.	Falling intonation
,	Level or slight rise intonation
?	High rising intonation

#### Data sessions

Analytic credibility was provided by studying these CA transcripts in *data sessions* in which linguists and CA experts and two GPs, an internist-oncologist, a spiritual caregiver and a patient participated [25]. The analysis always started with the patient’s lived illness experience, and then the oncologist’s response followed by that of the patient. One of the oncologists with expertise in research gave feedback on the findings.

### Ethical considerations

The study was performed according to the Dutch law and Good Clinical Practice guidelines [26, 27]. The Medical Review Ethics Committee region Arnhem-Nijmegen concluded this study was not subject to the Medical Research Involving Human Subjects Act (case number CMO:2018–4992/date 28 December 2018). In addition to the written consent previously given, all patients and their family caregivers also gave verbal informed consent prior to the observations and audio-recordings. All data were stored and analysed anonymized.

## Results

**Fig. 1:**
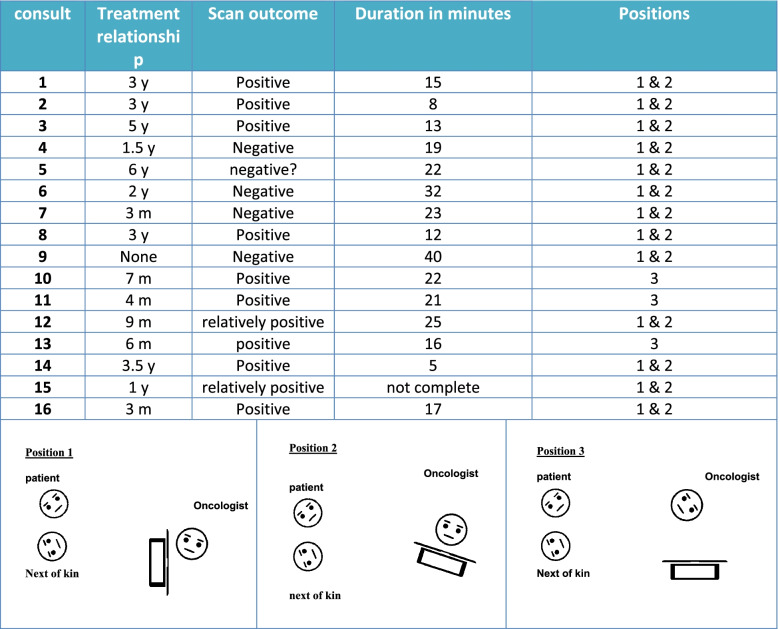
Consultation characteristics

Sixteen outpatient consultations with ten medical oncologists and 16 patients were observed and recorded. There were three refusals to participate: one oncologist refused and two patients were unwilling to participate. The ten oncologists (seven women) had work experience ranging from 2 to 35 years. The treatment involved different types of cancer: breast, ovary, skin, salivary gland, soft tissue, head/neck, thyroid, bladder, kidney, and prostate. Details of the consultations are shown in Fig. 1.

We identified 37 fragments in which patients expressed a lived experience, from 11 of the 16 consultations. In 13 fragments the response of the oncologist invited the patient to talk about their lived illness experience further, while in 24 fragments the response of the oncologist prevented him or her from doing so.

Oncologist responses that encouraged patients to tell more about these experiences were: producing a continuer such as humming, or trying to capture the raised lived illness experience. Oncologists responses with optimistic talk or presenting medical evidence did not result in inviting patients to further unfold their experience.

In four of the 21 fragments in which oncologist responses stopped further unfolding, the patient came back to it later. And twice, a patient did not elaborate further on their lived illness experience after the oncologist tried to put this experience into his own words; in both cases, this response was immediately followed by a presentation of medical evidence. The main types of responses are presented below.

Almost all oncologists were seated in front of their computer during the consultations (position 1). At some point, most oncologists turned the computer screen to show the scan image to the patient and relative (position 2). In three consultations the screen and keyboard were pushed aside and not used at all (position 3).

### Patients elaborate on their lived illness experience

In 13 of the 37 fragments, patients elaborated on their lived illness experience. This was preceded by oncologist responses such as trying to capture the lived illness experience of the patient in their own words, producing a continuer like a single short word such as ‘yes’, making an exclamation like ‘oh dear’, or humming.

### Oncologist produces continuers

An example of an oncologist producing continuers in response to a patient’s raised lived illness experience can be found in Table 2*.* The husband also participated in this example. The oncologist was seated in front of the computer, making notes.

**Table 2:** Oncologist producing continuers

1.	O:	so in that respect I was very happy when I eh (.)
2.		saw the scan;
3.	P:	I’m very happy because I was worrying a bit and that
4.		IS;
5.		oh I’ll speak a bit more clearly;
6.		•h that’s in fact also because we have an acquaintance;
7.	O:	yes,
8.	P:	who recently died of lung cancer;
9.	O:	oh my,
10.	P:	and he had metastases he had;
11.	O:	yes;
12.	P:	and he had colon cancer but then even for you it’s: a
13.		bit worrying
14.	O:	yes;
15.	P:	[>you know<] I’ve had breast cancer so:
16.	N:	[(yes exactly)]
17.	O:	yes and he’ll have had metastasis of the colon cancer in=
18.		[his lungs]
19.	N:	[yes yes exactly]
20.	P:	[yes yes yes;]
21.	N:	that was the case=
22.	P:	=so then you [start] to think
23.	O:	[yes;]
24.		y↑es;
25.	P:	and he passed away last week so •h (0.3) we went to the
26.		funeral and so on and that’s- (0.5) really been lingering in my head
27.		I must [say;]

In line 3 of Table 2, the patient raises an experience relevant to her illness: *‘I’m very happy because I was worrying a bit….’* She then uses emphasis to make clear that it is important to her that the oncologist listens *(‘and that IS’* line 3 and 4) and by inserting that she will speak more clearly (line 5). She then talks about an acquaintance who recently died of colon cancer. The oncologist does not interrupt but uses continuers: *‘yes’* (line 7, line. 11, line 14) and an evaluative *‘oh my’* (line 9).

Then (line 12) the patient shows the connection between her acquaintance’s story and her own illness by saying: *‘and he had colon cancer but then even for you it’s a bit worrying.’ (line 12 and 13)* and: *‘you know, I’ve had breast cancer so..’.* (line 15). The oncologist then formulates how she interprets the patients’ story so far [28]*: ‘yes and he’ll have had metastasis of the colon cancer in his lungs?’* (line 17 and 18). The patient and next of kin confirm this formulation three times with a ‘yes’ (line 20) and the patient rephrases her worrying’: ‘*so, then you start to think*’. Again, the oncologist produces a continuer (‘*yes*’) whereupon the patient unfolds her lived illness experience by elaborating on her ‘worrying’: *‘and he passed away last week so, we went to the funeral and so on, and that’s really been lingering in my head, I must say.’* (lines 25–27).

### Oncologist tries to capture the lived illness experience in own words

When an oncologist responded to a raised lived illness experience by trying to capture this experience in his or her own words, the patient also elaborated on this experience. An example of this is shown in Table 3. In this consultation the husband also participated; the computer screen and keyboard were pushed aside. (Table 1 Position 3).

**Table 3:** Oncologist tries to capture the lived illness experience

1.	N:	yes yes;
2.		(0.3) SUper
3.	P:	yes super good;
4.		[((laughs))]
5.	A:	[yes:]
6.		and uh [()]
7.	P:	[boy] I had’nt dared to hope;
8.		(0.4) ((sniffs))
9.	A:	(0.7) no: it’s always worrying;
10.		that [you:]
11.	P:	[yes]
12.	A:	I did say that the CHANCE;
13.		(0.2) that it would respond [of]course was bigger than with the
14.		(*name drug*)
15.	P:	[yes]
16.		[that’s] right;
17.	A:	[but]
18.	P:	but I [thought yes: (.) and the blood] values of course have been
19.		>the whole time < extremely goo:d;
20.	A:	[*still always wait and*]…
21.		yes;
22.	P:	but I thought yes what is it doing inside there,
23.	A:	yes;
24.	P:	(0.5) and that’s what I was a > bit afraid of<;

Prior to the start of this extract, the medical oncologist had announced ‘good news’ from the scan. In lines 1–4 of Table 3, the husband and the patient welcome this news respectively with: ‘*Yes, yes, super*’ and *‘yes, super good’*. Subsequently, in line 7, the patient articulates an experience relevant to her illness: *‘Boy, I had not dared to hope’*. The oncologist does not respond immediately but as the patient does not elaborate further, the oncologist tries to capture her ‘*not dared to hope*’ in his own words by saying: *‘No, it’s always worrying, that you (…) I did say that the chance that it would respond of course was bigger than with the [name drug].’* (lines 9–14). After the patient, in line 15–16, confirms what the oncologist has outlined (*‘Yes, that’s right’*), she continues by talking more about her lived illness experience: *‘but I thought, yeah, and the blood values of course have been the whole time extremely good‘.* Again the oncologist responds by capturing in his own words the patient’s experience:*‘ still always wait and* [*see*]*…’.* (line 20) He adds an open ‘but’ which is immediately taken up by the patient and complemented by a further elaboration on her lived illness experience: *‘But I thought, yeah, what is it doing inside there’* (line 22). The oncologist then produces a continuer (‘*yes’* in line 23) and the patient elaborates further:*’…and that’s what I was a bit afraid of’*.

### Patients do not elaborate on their lived illness experience

In 24 of the 37 fragments, patients did not elaborate on their raised lived illness experience. Two forms of oncologist response preceded this: presenting medical evidence like findings drawn from statistics, radiographs or scans, or by talking optimistically like *‘Well, this all looks good’*. We found some overlap between these two forms of response: when presenting medical evidence, the discussion often was a moment of optimism. In four extracts in which the raised lived illness experience was not elaborated on, the patient did not withdraw it, but repeated it in (slightly) different terms.

### Oncologist presents medical evidence

Table 4 gives an example of an oncologist presenting medical evidence as a response to a raised lived illness experience of a patient. The patient’s husband was also present. The patient had recently been diagnosed with advanced cancer for which there was no effective treatment. The oncologist was seated in front of the computer and made notes. Prior to this extract, the oncologist had tried to end the conversation, suggesting calling the patient the following Wednesday. The patient snivels and raises a thought relevant to her illness: ‘*And how, how long do I still have? It won’t be long, it won’t be long anymore I think*’ (Table 4, line 2–4). In her response, the oncologist presents medical evidence beginning with: ‘*No, that’s what I think too*’ (line 5), followed by: *‘But I really think that if I thought that we with immunotherapy, that it really could beautifully lengthen it, I would start tomorrow. But I think that we that, because … we can’t do that’* (line 7–12). The oncologist continues her reasoning for not giving immunotherapy, based on medical evidence. The patient’s lived illness experience regarding *‘how long do I still have?’* does not further unfold.

**Table 4:** Oncologist presents medical evidence

1.	P:	((sniffs))
2.		(2.5) ((cries)) and how (.) how long do I still have, ((cries))
3.		(2.0) ((cries)) it won’t be long, ((cries))
4.		(3.9) ((cries)) it won’t be long anymore I think ((cries))
5.	A:	no that’s what I think too;
6.	P:	((sniffs))
7.	A:	(3.2) but I re- really think that if I thought that we with
8.		immunotherapy, that it really(.4) could beautifully lengthen it,
9.		I would start tomorrow;
10.		(2.5) but I think that we tha-
11.		((coughs))
12.		because (.) we can’t do that;

### Oncologist talks optimistically

Another response restraining patients from elaborating on their lived illness experience was optimistic talk by the oncologist, as can be seen in Table 5. In the consultation from which this fragment is taken, the patient’s wife was also present. The oncologist was seated in front of the computer and made notes. A few months before this consultation, a new treatment had been started that proved effective. Prior to this fragment, the patient told the oncologist about a new study he had read about.

**Table 5:** Oncologist talks optimistically

1.	A:	[so] we eh: we are eh: with our nuclear colleagues we are •h
2.		tryin’ to b- a combination of those two substances eh: to
3.		(0.4) eh: (.) administer;
4.		(0.6) •pt. in a in a study;
5.	P:	yes:yes;
6.	A:	>yes that that, hg we can [hopefully in the-]
7.	P:	[well I hope I’ll] make it;
8.	A:	•h well,
9.		(0.5) this looks- so far all looks good so that: that’s
10.		right?
11.	P:	yes: [well as long as this] is good,
12.	A:	[now it’s starting to work,]
13.	P:	yes well well!;
14.	A:	•h it can be that eh: that eh this treatment,
15.		eh: (.) it will work for (0.3) one and a half years [he so that eh: if it]
16.	P:	[h↓m: if it]
17.		works well but [in the meantime]
18.	A:	[if it works well;]
19.		(0.5) so we’ll keep’ [all options:]
20.	P:	[a lot is happening] still;
21.	A:	exactly;
22.		exactly;
23.		[op- we’ll] keep all options open,
24.	N:	[yes:;]
25.	A:	•h and eh: they‘r indeed many new treatments coming up;
26.		the psma is one of them,
27.		but: also one is coming one •h one treatment with olaparib (.) one
28.		different (0.2) new (0.5) drug which maybe is just as good
29.		and maybe even better than that psma-therapie,
30.	P:	hmm,
31.	A:	so a lot of new things are coming up so;
32.		•h
33.	P:	yesyes;
34.	A:	that’s eh;
35.	P:	We’ll wait for them and I hope I’ll still make it;
36.		so eh: he,
37.	A:	yes;
38.		•h okay;
39.		and briefly (0.4) back to eh: the order of the day [>because we:<]
40.	P:	[yes]

In line 1–6 of Table 5 the oncologist resumes his previous answer, using words that point to the future: ‘*trying*’, *‘to administer’*, *‘in a study’* and *‘hopefully*’. The patient responds by raising an experience relevant to his illness: ‘*Well, I hope I’ll make it’* (line 7). The patient repeats this experience twice more, in slightly different words: ‘*yes, well as long as this is good’* (line 11) and ‘*ahem, if it works well, but in the meantime….’* (line 16–17).

The oncologist responds repeatedly by talking optimistically: *‘Well, this looks, so far, all looks good, so that, that’s right?*’ (line 8–10), ‘*now it’s starting to work’* (line 12), *‘so we’ll keep all options[open]’* (line 19). In line 20, the patient aligns with the oncologist by briefly describing what he has understood: *‘a lot is happening still‘*. The doctor confirms his summary by once again repeating that all options will remain: *‘exactly, exactly, we’ll keep all options open’* and responds once again by optimistically discussing future options (lines 25–29). The patient now repeats his lived illness experience: *‘we’ll wait for them and I hope I’ll still make it; so eh ...’* (line 35–36). In his response the oncologist asks the patient to move on to a next topic by saying: ‘*Yes. Okay. And briefly back to eh, the order of the day’*.

## Discussion

This study provides insights into whether and how medical oncologists at an outpatient clinic identify and explore lived illness experiences raised by patients with incurable cancer. We note two forms of oncologists’ responses to these experiences that encouraged patients to elaborate on them: by producing ‘continuers’ or by trying to capture the patients’ experience in their own words. Patients did not elaborate on their experiences in cases where oncologists responded with optimistic talk or presented medical evidence.

In most of the observed consultations, patients raised lived illness experiences. This is in line with previous studies that showed that patients on the oncology ward directly or indirectly express what mostly concerns them [13]. Weiner et al. denote listening to what matters as efficient healthcare provision ‘*because it uncovers the actual underlying issues that account for the presenting problem*’ [29]. Overlooking the patient’s life context may even result in inappropriate and costly care [17].

However, oncologists often find it challenging to talk to patients about their illness, life and death [3, 30]. For patients, a hospital is often an environment in which it can be a challenge to share their concerns, experiences and questions [31]; they follow their doctors’ optimistic talk, although they often regret this later [32, 33].

Interestingly, in those conversations where the patient elaborated most on the lived illness experience, the computer screen and keyboard were pushed aside. Earlier research shows that physicians’ simultaneous use of a computer and speech compromises communication skills [34], and that the computer is often a distracting source of information [35]. Moreover, when attention was paid to the lived illness experience, the consultations did not take any longer than those where no attention was paid, which is in line with previous findings [17].

### Study strengths and limitations

This is the first study to examine, during live outpatient consultations, whether and how patients’ lived illness experiences occur, how medical oncologists respond, and the effects. The multidisciplinary approach to the analysis resulted in a wide range of relevant viewpoints. The CA analysis proved valuable in capturing the real time interaction of patient-oncologist consultations.

However, all interviews were recorded at the same university medical centre, from one team of oncologists. Also, the fact both oncologists and patients were informed about the subject of the study may have caused bias.

Although nearly 40 extracts were included, these originated from 11 of the 16 consultations, conducted by nine oncologists. Differences between oncologists regarding sex, age and experience were not included in our analyses. Although earlier research did not find any significant influence of those variables on communication outcomes [36], we do recommend including such characteristics in future studies. Furthermore, for each patient, we only recorded one of a series of consultations, not knowing what had already been discussed at these other consultations, which limits its generalisability. Moreover, our analyses did not capture how conversations created conditions to actually express lived illness experiences.

Lastly, although video recordings would have made it possible to also fully include non-verbal communication in the analyses, audio recordings were chosen in order to prevent patients from feeling uncomfortable.

### Clinical implications

According to CanMEDS (Canadian Medical Education Directions for Specialists), the most widely accepted and applied physician competency framework in the world, being a ‘communicator’ is one of the 8 core skills of physicians [37]. However, research shows that there are many shortcomings and pitfalls in the daily communication between physicians and patients [18]. We believe our findings make a valuable contribution to bridging the gap between reluctance of both oncologists and patients when discussing illness, life and death. Our findings are not complex, and easily applicable. It requires medical oncologists to be more alert to raised lived illness experiences of patients and to actively listen to patients who spontaneously introduce these issues. Yet, it is difficult to change established practice, and ‘culture eats strategy for breakfast’. Addressing and integrating the lived illness experience in consultations does not have to take extra time, and may even contribute to more efficient consultations [17]. However, paying attention to and acquiring (more) competence in exploring the lived experiences may demand training sessions with, for example, simulation patients or coaching on the job [36]. Moreover, we recommend ‘frequent reinforcement’, for example during patient handovers or case discussions [29].

## Supplementary Information

**Additional file 1.** COREQ checklist.

## Data Availability

The data that support the findings of this study are available on request from the corresponding author. The data are not publicly available due to privacy or ethical restrictions.

## Abbreviations

WHO: World Health Organization
JAMA: Journal of the American Medical Association
CA: Conversation Analysis
GP: General practitioner
