# Correction: Oncologist responses to advanced cancer patients’ lived illness experiences and effects: an applied conversation analysis study

**DOI:** 10.1186/s12904-022-00969-6

**Published:** 2022-05-12

**Authors:** Jacqueline van Meurs, Wyke Stommel, Carlo Leget, Joep van de Geer, Evelien Kuip, Kris Vissers, Yvonne Engels, Anne Wichmann

**Affiliations:** 1 Department of Spiritual and Pastoral Care & Department of Anesthesiology, Pain and Palliative Medicine, Radboud University Medical Centre, P.O. Box 9101 (714), 6500 Nijmegen, HB Netherlands; 2 Centre for Language Studies, Radboud University, Nijmegen, the Netherlands; 3 Department of Care and Welfare, University of Humanistic Studies, Utrecht, The Netherlands; 4 Academic Hospice Demeter, Bilthoven & Agora, De Bilt, the Netherlands; 5 Department of Medical Oncology & Department of Anesthesiology, Pain and Palliative Medicine, Radboud University Medical Centre, Nijmegen, the Netherlands; 6 Department of Anesthesiology, Pain and Palliative Medicine, Radboud University Medical Centre, Nijmegen, the Netherlands

**Correction to: BMC Palliative Care 21, 1-9 (2022)**

**https://doi.org/10.1186/s12904-022-00917-4**

Following the publication of the original article [1], the author reported that in Tables 3, 4 and 5, instead of an 'A' for Oncologist, it should be an 'O'

The original article has been updated.
